# Supplementary material for: Identification of photoperiod-induced specific miRNAs in the adrenal glands of Sunite sheep (Ovis aries)
Source: Front Vet Sci. 2022 Jul 22;9:888207. doi: 10.3389/fvets.2022.888207 (PMC9354845; doi:10.3389/fvets.2022.888207)
Supplement: Supplementary file 1 [file Data_Sheet_1.ZIP › Supplementary file/miRDeep2 file.zip!/miRDeep2 file/Know_Structure/LP42A3/oar-mir-3958.pdf]

miRBase precursor : oar-mir-3958
Total read count : 24623
oar-miR-3958-5p read count : 27
oar-miR-3958-3p read count : 24592
remaining reads : 4

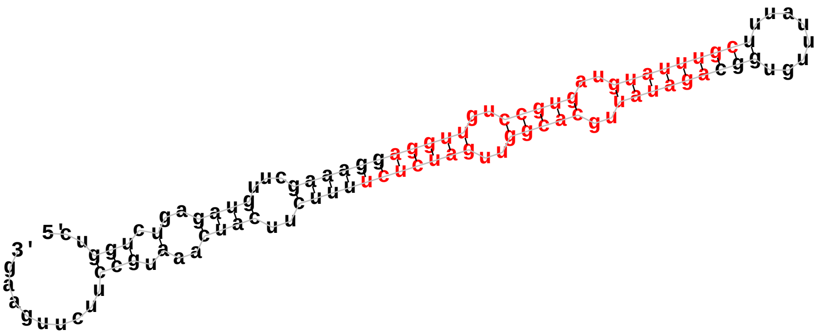

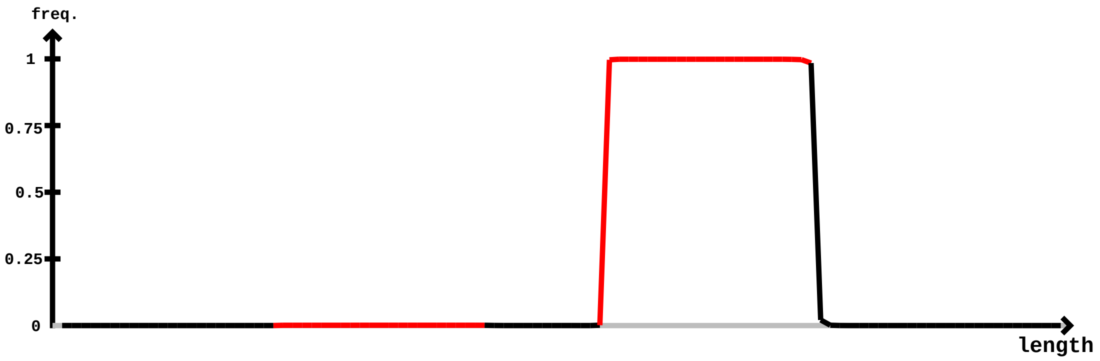

oar-miR-3958-3p

oar-miR-3958-5p

```
5'- cuggucugagauguucgaaaggagguuguccgugauguauuugcuuuauuuguggcagauauugcacgguugaucucuuuucuucaucaaaugccuucuugaag  -3'   exp
    ..(((.(..((((...((((((((((..(((((..(((((((((........)))))))))..)))))..)))))))))))..)))).).))).........   reads   mm   sample
    cuggucugagauguucgaaagg..................................................................................   2       0    seq
    ......................agguuguccgugauguauuug.............................................................   3       0    seq
    ......................agguuguccgugauguauuugc............................................................   17      0    seq
    ......................agguuguccgugauguauuugcu...........................................................   7       0    seq
    ......................agguuguccgugauguauuugcuuuauuugu...................................................   1       0    seq
    ..............................cgugauguauuugcuuuauuuguggc................................................   1       0    seq
    .......................................................cagauauugcacgguugaucu............................   1       0    seq
    .......................................................cagauauugcacgguugaucuc...........................   1       0    seq
    .......................................................cagauauugcacgguugaucucu..........................   31      0    seq
    ........................................................agauauugcacgguugauc.............................   13      0    seq
    ........................................................agauauugcacgguugaucu............................   27      0    seq
    ........................................................agauauugcacgguugaucuc...........................   314     0    seq
    ........................................................agauauugcacgguugaucucu..........................   23634   0    seq
    ........................................................agauauugcacgguugaucucuu.........................   483     0    seq
    ........................................................agauauugcacgguugaucucuuu........................   28      0    seq
    ........................................................agauauugcacgguugaucucuuuu.......................   5       0    seq
    .........................................................gauauugcacgguugaucucu..........................   53      0    seq
    ..........................................................auauugcacgguugaucucu..........................   1       0    seq
    ...........................................................uauugcacgguugaucucu..........................   1       0    seq
```
